# Intensive Case Finding and Isoniazid Preventative Therapy in HIV Infected Individuals in Africa: Economic Model and Value of Information Analysis

**DOI:** 10.1371/journal.pone.0030457

**Published:** 2012-01-23

**Authors:** Hendramoorthy Maheswaran, Pelham Barton

**Affiliations:** 1 Division of Health Sciences, University of Warwick Medical School, Coventry, United Kingdom; 2 Health Economics Unit, School of Health and Population Science, University of Birmingham, Birmingham, United Kingdom; San Francisco General Hospital, University of California San Francisco, United States of America

## Abstract

**Background:**

Tuberculosis (TB) accounts of much of the morbidity and mortality associated with HIV. We evaluate the cost-effectiveness of different strategies to actively screen for TB disease in HIV positive individuals, where isoniazid preventative therapy (IPT) is given to those screening negative, and use value of information analysis (VOI) to identify future research priorities.

**Methodology/ Principal Findings:**

We built an individual sampling model to investigate the costs (2010 US Dollars) and consequences of screening for TB, and providing TB treatment or IPT in adults testing HIV positive in Sub-Saharan Africa. A systematic review and meta-analysis was conducted to assess performance of the nine different TB screening strategies evaluated. Probabilistic sensitivity analysis was conducted to incorporate decision uncertainty, and expected value of perfect information for the entire model and for groups of parameters was calculated. Screening all HIV infected individuals with sputum microscopy was the least costly strategy, with other strategies not cost-effective at WHO recommended thresholds. Screening those with TB symptoms with sputum microscopy and CXR would be cost-effective at a threshold ICER of $7,800 per quality-adjusted life year (QALY), but associated with significant uncertainty. VOI analysis suggests further information would be of value.

**Conclusions/ Significance:**

Resource-constrained countries in sub-Saharan Africa wishing to scale up TB preventative services in their HIV infected populations should consider expanding laboratory facilities to enable increased screening for TB with sputum microscopy, whilst improved estimates of the TB prevalence in the population to be screened are needed, as it may influence the optimal strategy.

## Introduction

Tuberculosis (TB) and human immunodeficiency virus (HIV) together pose a immense challenge to health services in sub-Saharan Africa, where over 22 million people are living with HIV [1]. TB is the commonest cause of mortality and morbidity in HIV infected individuals [2], [3], even after initiation onto highly active antiretroviral treatment (HAART) [4], [5]. Whilst the incidence of TB is lower in those on HAART than those not on HAART [6], [7] and falls with time on HAART [4], [8], it still remains higher than that found in HIV negative populations [9]. Importantly, the higher incidence of TB in the initial months of initiating HAART [4], [8] is felt partly to be a consequence of active TB not being detected through routine screening [10].

HIV infected individuals are at increased risk of active TB as a consequence of reactivation of latent TB infection (LTBI) [3], [11] or from recent infection with Mycobacterium tuberculosis rapidly progressing to active disease. Isoniazid preventative therapy (IPT) reduces this risk, which in combination with increased case detection of active TB is essential to impact on TB control and improve outcomes in HIV positive individuals [12], [13]. In view of this the WHO's “Three Is” policy of intensified case finding (ICF), IPT, and infection control, recommends all individuals testing HIV positive in sub-Saharan Africa be screened for active TB disease, and if no evidence found, receive IPT with or without prior tuberculin skin testing [14]. Despite this only 9% of HIV infected individuals are actively screened and 3% offered IPT [15], partly as a consequence of the resource requirements needed to implement these interventions [16].

There has been considerable research comparing the diagnostic accuracy of different TB screening strategies [17]–[26], but still no clear consensus on the most appropriate approach for ICF that takes into account both the decisions of starting TB treatment or initiating IPT. ICF and IPT have both been shown to be a cost-effective intervention in sub-Saharan Africa [27]–[31], however, previous studies have not considered the combined costs and consequences of implementing both interventions in conjunction, nor investigated alternate screening strategies. We therefore sought to evaluate the cost-effectiveness of simple TB screening strategies that could be implemented, where HIV positive individuals are either diagnosed with TB or started on IPT. We evaluate a variety of strategies for which comparative cost-effectiveness data is presently unavailable and for which naturalistic trials would be difficult to undertake, and use value of information (VOI) analysis to identify future research priorities [32].

## Methods

### Analytic Overview

We used an individual sampling model (ISM) to evaluate the incremental cost-effectiveness of nine TB screening strategies. An ISM was chosen to track individuals' histories and keep the model structure to a manageable size, which would not have been possible under the homogeneity assumption of Markov models [33]. The model evaluated a hypothetical population of individuals aged over 16 years testing HIV positive at health centres in sub-Saharan Africa. Cost per quality adjusted life year (QALY) was the primary outcome, and costs adjusted for inflation to 2010 US Dollars using a gross domestic product (GDP) deflator [34], as recommended for economic evaluations in resource-constrained settings [35]. The time horizon was two years, the duration of benefit of IPT [36], and cycle length one month. The public health provider perspective was chosen, and an annual discount rate of 3% applied to both costs and health effects [35]. The model was built using TreeAge Pro 2009 (TreeAge Software, Williamstown, MA).

### Screening strategies

**Figure 1:**
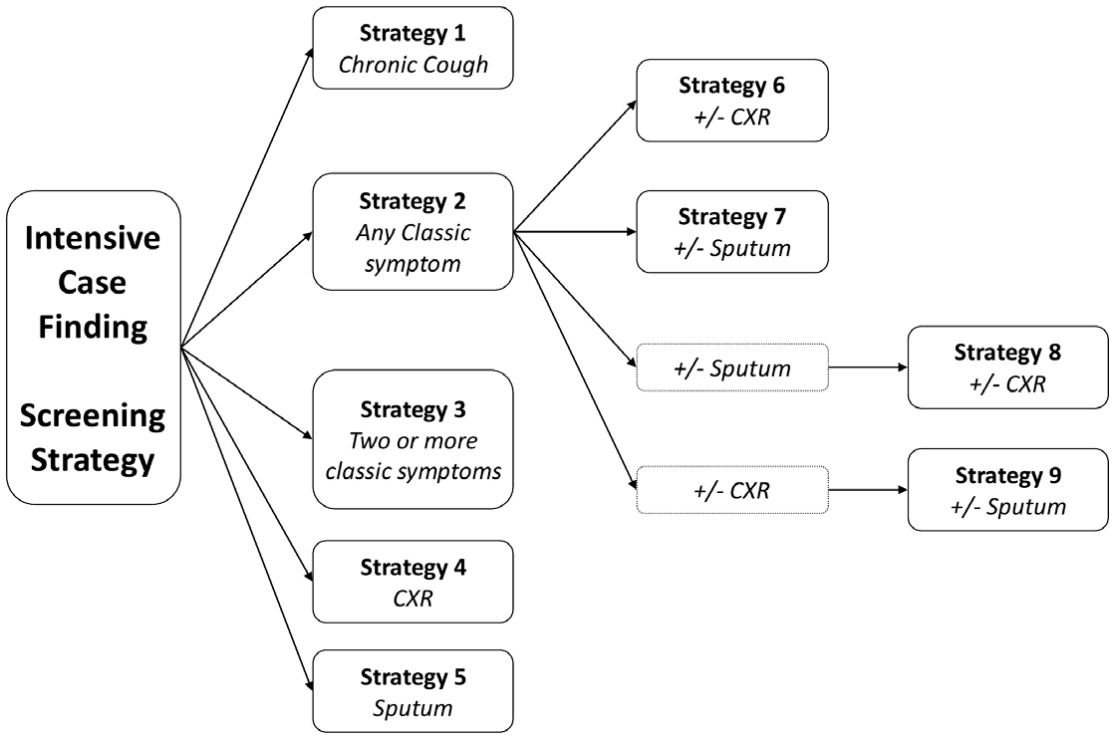
Description of the nine screening strategies.

Figure 1 shows the nine TB screening strategies investigated. The strategies use either one or a combination of: symptom screening; sputum microscopy for acid-fast bacilli; or chest radiography (CXR). For symptom screening the classical symptoms of TB (cough, fever, night sweats or weight loss) are commonly reported among TB patients [21], [25]. We investigated screening for the presence of: chronic cough (Strategy 1); any one of the classical symptoms (Strategy 2); or two or more of the classical symptoms (Strategy 3). Four strategies used either CXR or sputum microscopy. We considered screening all individuals irrespective of whether they had any TB symptoms, (Strategies 4 [CXR] and 5 [sputum microscopy]). We also considered screening only individuals with TB symptoms (TB suspects), (Strategies 6 [CXR] and 7 [sputum microscopy]). Finally, we considered strategies using both CXR and sputum microscopy in TB suspects. Sputum microscopy could be performed in all TB suspects and if negative CXR performed (Strategy 8); conversely, CXR could be performed first and sputum microscopy in those with negative findings on CXR (Strategy 9).

### The Model

**Figure 2:**
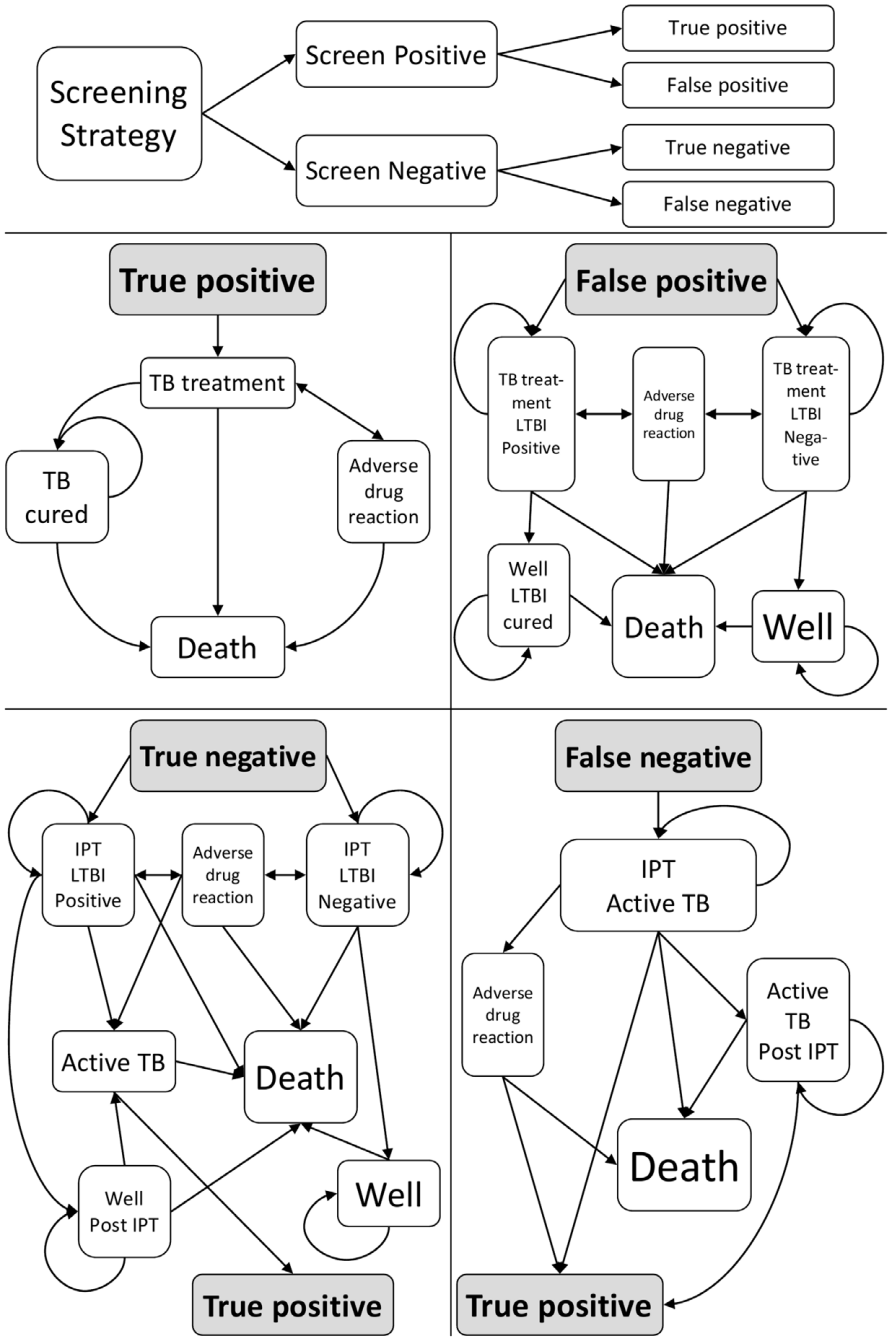
Description of models built.

Figure 2 describes how the screening strategy classifies individuals into one of four groups, those screening positive who either had TB disease (true positive) or not (false positive), and those screening negative who either did not have TB disease (true negative) or had TB disease (false negative). A Markov model then simulates treatment related costs and outcomes for the four groups (Figures S1, S2, S3, S4). Those without active TB, true negatives and false positives, may or may not have LTBI. We therefore used separate health states to account for their differing utilities and subsequent risk of developing active TB.

Individuals screening positive for TB were given 6 months of anti-TB treatment and those screening negative were given 6 months of Isoniazid (INH) [37]. The adverse consequences of treatment were also modelled [38]. Those with active TB who successfully completed TB treatment were assumed not to be at risk of recurrence in the immediate future. Those falsely started on TB treatment (false positive) were assumed not to be at risk of developing TB either during or after treatment. Those correctly excluded for active TB (true negatives) but who had LTBI were assumed to be at risk of developing active TB during their IPT course, their risk reduced after completion of IPT [39], whilst those without LTBI were assumed not to be at risk of developing active TB. Individuals who had active TB not diagnosed (false negative) experienced the disutility of active TB disease, but we allowed for the possibility that they might seek care later. We assumed that those who sought care later would undergo the same screening strategy and the outcome of screening was independent of an individual's prior screening outcome. Individuals with active TB were at risk of TB related mortality, until completion of TB treatment, whilst all individuals were at risk of non-TB related mortality. The costs and benefits of HAART were not considered and we assumed there was no HIV disease progression, during the 2 years the model was run.

### Input parameters for Model

#### Data sources

We carried out several formal systematic reviews to identify model parameters, and limited our search to studies done in resource-constrained settings. We performed a meta-analysis of data extracted if possible, if not we used data from the most recent meta-analysis, and if not present we used data from relevant studies. Table 1 shows the input parameters used in the model. The low and high values represent the reported 95% confidence interval, when not available they represent the lowest and highest values from the range of studies found.

**Table 1:** Parameters used in model.

Parameter	Base	Low	High	Distribution	Source
**Transition probabilities**					
**Strategy 1:** Chronic cough>2 weeks					
Sensitivity	0.440	0.399	0.482	Beta	[17]–[26]
Specificity	0.810	0.802	0.818	Beta	
**Strategy 2:** Any Classic symptom					
Sensitivity	0.889	0.860	0.913	Beta	[17], [19]–[26]
Specificity	0.492	0.482	0.502	Beta	
**Strategy 3:** Two or more Classic symptoms					
Sensitivity	0.415	0.361	0.470	Beta	[18], [22], [23], [25]
Specificity	0.904	0.892	0.915	Beta	
**Strategy 4:** CXR on all					
Sensitivity	0.635	0.586	0.682	Beta	[18], [19], [22], [23], [25], [26]
Specificity	0.865	0.855	0.875	Beta	
**Strategy 5:** Sputum on all					[17]–[19], [22], [25]
Sensitivity	0.403	0.354	0.454	Beta	
Specificity	0.990	0.986	0.993	Beta	
**Strategy 6:** Symptom +/− CXR					
Sensitivity	0.684	0.581	0.776	Beta	[19], [22]
Specificity	0.800	0.785	0.815	Beta	
**Strategy 7:** Symptom +/− Sputum					
Sensitivity	0.439	0.387	0.492	Beta	[19], [22], [25]
Specificity	0.880	0.870	0.890	Beta	
**Strategy 8:** Symptom +/− Sputum +/− CXR					
Sensitivity	0.672	0.616	0.725	Beta	[23], [25]
Specificity	0.867	0.851	0.882	Beta	
**Strategy 9:** Symptom +/− CXR +/− Sputum					
Sensitivity	0.7580.791	0.659	0.840	Beta	[19], [22]
Specificity		0.776	0.806	Beta	
TB prevalence	0.086	0.036	0.247	Beta	[40]
LTBI prevalence	0.31	0.27	0.46	Beta	[41]
Seeking care – Passive case finding	0.1985	0.0779	0.4512	Beta	[77]
Adverse event on TB treatment	0.0032	0.0023	0.0044	Beta	[78]
Fatal event on TB treatment given adverse event	0.0504	0.0033	0.1552	Beta	Estimated
Adverse event on IPT	0.0023	0.0008	0.0090	Beta	[60], [79]–[81]
Fatal event on IPT given adverse event	0.0360	0.0009	0.1277	Beta	[60]
Risk of TB in those with LTBI: Pre-completion of IPT	0.0057	0.0050	0.0066	Beta	[43]
Post-completion of IPT	0.0009	0.000017	0.0061	Beta	[43]
Death in treated TB patients	0.0145	0.0047	0.0246	Beta	[39]
Death in untreated TB patients	0.0488	0.0392	0.0582	Beta	[82], [83]
Death from non-TB	0.00022	0.000067	0.00078	Beta	[44]
**Cost Parameters (2010 US Dollars)** *					
TB treatment**	$78.97	$60.42	$111.74	Gamma	[30], [84], [85]
IPT**	$21.08	$10.55	$31.63	Gamma	[30]
Adverse effects of IPT and TB treatment**	$344.95	$115.22	$566.74	Gamma	[30], [86]
Strategy 1–3	$3.08	$1.43	$9.51	Gamma	[45]
Strategy 4	$16.07	$11.74	$43.91	Gamma	[45]
Strategy 5	$5.24	$2.90	$12.28	Gamma	[45]
Strategy 6	$15.14	$10.23	$42.44	Gamma	[45]
Strategy 7	$7.02	$3.62	$18.72	Gamma	[45]
Strategy 8	$13.45	$8.30	$35.24	Gamma	[45]
Strategy 9	$17.24	$11.40	$47.35	Gamma	[45]
**Utility Parameters**					
Stage 2: -Well	0.63	0.60	0.65	Beta	[47]
-LTBI/Post LTBI treatment	0.56	0.53	0.59	Beta	Estimated
-Post TB treatment	0.49	0.46	0.52	Beta	Estimated
Stage 3: -Active TB disease	0.39	0.37	0.42	Beta	[47]
Stage 4: -Adverse effects of TB/LTBI drugs	0.15	0.13	0.17	Beta	[47]
**Parameter**	**Base**	**Low**	**High**	**Distribution**	**Source**

*Costs for screening strategies based on: Health clinic visit, $3.08 (1.43–9.51); Outpatient clinic visit, $4.61 (1.43–31.30); Health worker daily salary, $23.52 (17.59–56.62); Nurse daily salary, $33.92 (25.65–82.90); Physician daily salary, $57.12 (42.74–137.77); Sputum microscopy $2.56 (1.78–3.25); CXR $11.46 (10.31–12.61).

**Total program cost inclusive of overheads, personnel and drugs. For TB treatment; low value represents Community-based care with up to 19 days hospitalization [85]; base case value [85] and high value [84] represent hospital-based care with up to 60 days hospitalization.

#### Transition probabilities

Sensitivities and specificities of the TB screening strategies were pooled using Meta-DiSc version 1.4 (Unit of clinical biostatics, the Ramo y Cajal Hospital, Madrid, Spain). Random effects model was used to pool values because of significant between-study heterogeneity. The prevalence of TB in HIV populations was taken from a recent meta-analysis [40], whilst the prevalence of LTBI in sub-Saharan Africa was extracted from a study done by Corbett et al [41]. There was no data available on the risk of a fatal event in those who experienced adverse events on TB treatment. As the treatments have similar side effect profiles [42], we assumed that the ratio of the risk of fatal events, subsequent to an adverse event, with TB and LTBI treatment was proportional to the ratio of the risk of adverse events with TB and LTBI treatment. We found no applicable data regarding uptake and subsequent loss to follow-up (LTFU) from ICF and IPT programmes. We therefore assumed all those screened would initiate and complete their respective treatments, however, this was further investigated in the sensitivity analysis. The risk of TB in those with LTBI, pre- and post- IPT, was extracted from a recent study by Golub et al [43]. As a consequence of lack of relevant data we assumed that TB and non-TB related mortality in individuals with early HIV disease not on HAART is comparable to mortality in individuals with more advanced HIV disease but receiving the benefits of HAART. The risk of TB related mortality for individuals on TB treatment was extracted from a recent meta-analysis [39]. For non-TB related mortality in HIV populations, data was extracted from a large study that presented findings from a number of HIV treatment centres across sub-Saharan Africa [44]. The mortality rate for those in whom HAART had been initiated with early HIV disease and who survived the first year of treatment was used. We assumed that this risk of death included the risk from non-HIV related causes, as this was the endpoint in their study.

#### Costs of screening and care

The direct medical costs accrued by the organisation administering the screening, providing TB treatment or IPT, and managing adverse effects of treatment were considered. We assumed the screening strategy would be integrated within existing HIV and TB services at no additional start-up costs as these services are already in existence. However, we considered there would need to be some scale-up of services in terms of staffing required to implement the program. The cost of the screening strategies was derived from data published by the Disease Control Priorities Project (DCPP), a description of methods used has been published [45], and are in accordance with WHO costing guidelines [35]. Costs for the World Bank sub-Saharan Africa region were used to aid generalisability of findings.

The cost of the screening strategy included cost of a clinic visit and the additional cost of any investigations. The cost of a clinic visit consisted of the long-run capital costs and labour costs (15 minutes of healthcare worker and 15 minutes of a registered nurse). For investigations, costs include overhead, equipment, consumables and labour [45]. For strategies where sputum smears were done, costs were calculated for two specimens as the value of the third sputum is low [46]. For strategies incorporating a CXR the cost of an outpatient visit in a secondary care hospital and 15 minutes of a physician labour cost instead of a nurse was used to reflect the need for a clinician to interpret the film. For strategies involving a combination of screening methods, the cost of the whole algorithm was calculated by adding the cost for each screening step. As only a proportion would proceed to the next screening method, only a proportion of the cost was added. We assumed 75% of participants would proceed onto subsequent screening method if suspected and not diagnosed; this was based on attrition rates reported by Shrestha et al [30]. To ensure comparability between costs of providing TB treatment and IPT, and associated adverse events, data was extracted from the same study and represent the full program costs inclusive of drugs, labour and overheads [30]. The cost of TB treatment quoted was for an 8-month course. The WHO recommends a course of 6 months duration, as the additional two months provide no benefit [37]. We assumed that the monthly cost was constant and limited all individuals to 6 months treatment. There was no published data available for the costs associated with adverse reactions to TB treatment. As IPT and TB treatment have similar side-effect profiles [42], we assumed the costs to be equivalent.

#### Health related Quality of life

We used utility scores from data published by Lara et al [47] for their pre-HAART cohort, as this is considered the only valid utility data presently available [48]. We classified individuals based on their WHO HIV clinical stage [49], as was done in previous modelling exercises in this area [27], [30]. Utility scores associated with experiencing an adverse reaction to TB or LTBI treatments are similar [42] and have previously been assumed to be equivalent to WHO stage 4 [27], [30]. We made assumptions for utility scores for states associated with LTBI and post LTBI and TB treatment using evidence from the literature. Individuals with active TB have lower utility scores than individuals with LTBI [50], [51], and improve with treatment [51]. Individuals with LTBI have lower utility scores than those never previously infected [51], and show no change subsequent to treatment. The base case values assumed were chosen to reflect this and ranges for probabilistic analysis chosen to ensure no overlap with utility values for states preceding or arising from it, and wide to reflect the uncertainty underlying these assumptions.

#### Sensitivity analysis and Value of Information

Probabilistic sensitivity analysis (PSA) was performed using Monte Carlo simulation (MCS) to take into account uncertainty in individual patient outcomes and uncertainty in parameters values [52]. For each of the strategies we ran 10,000 model runs, with 10,000 individuals sampled for each run. Distributions used to represent parameter uncertainty were Beta for transition probabilities and utilities, and Gamma for costs [52]. VOI analysis was used as an alternative to univariate sensitivity analysis, and as an adjunct to PSA [53], to quantify the value of future research.

Determining the value of future research in resource-constrained settings provides an approach to aid efficient use of limited research funding and direct research to answering decisions faced by policy makers. PSA describes the uncertainty surrounding the optimal decision and indirectly the probability that the decision based on current information will be wrong. There are consequences of making the wrong decision, either in resources or health benefits forgone, which would be eliminated if we had perfect information. However, further information comes at a cost and is only valuable if it alters the optimal decision. VOI quantifies the expected value of perfect information (EVPI) by calculating the expected payoff if we had perfect information compared to existing information [54]. The EVPI will be greater when there is greater uncertainty around the optimal decision and when for a given level of uncertainty our maximum willingness to pay (WTP) threshold is higher [32], [55]. If the EVPI is greater than the cost of obtaining new information, then further research will be valuable. However, EVPI analysis on its own does not indicate what further information to obtain. To direct future research requires expected value of perfect parameter information (EVPPI). EVPPI analysis can be performed for any single parameter or group of parameters within a model and is used to calculate how much of the expected payoff, the expected value of perfect parameter information (EVPPI), would come from having perfect information on each of the selected input parameters. For computational reasons we performed a one-level MCS with the parameters of interest kept constant, whilst the remaining were sampled from their distribution [32]. The parameters were grouped based on how future research might be carried out. We used Pearson's correlation coefficient to investigate for the presence of significant correlations between input parameters, and performed multiple linear regressions for each of strategies' net monetary benefit as the dependent variable and the sampled input parameter as the independent variable. Provided that the randomly sampled input parameters are not significantly correlated and the net benefit is a multi-linear function of the parameters a one-level MCS can be performed [56], [57].

#### Sensitivity analysis: Alternative model structures

We investigated the impact of loss to follow up (LTFU) on the optimal strategy. Evidence suggests a significant proportion of patients starting either IPT or TB treatment may be LTFU prior to starting treatment, during the initial phases of treatment, or during the latter stages of treatment [58]–[62]. Completion rates range from 55% to 86% [43], [60], [62], [63] and 80% to 88% [58], [59], [61], respectively. We therefore assumed the risk of LTFU prior to initiating therapy was equal to that subsequent to initiating therapy. However, we assumed that this risk could differ between those starting IPT and those starting TB treatment. We also assumed those LTFU who had active TB could subsequently seek care and those who had LTBI were at risk of developing active TB. We ran the model investigating a range of completion rates, 100%, 80%, 60% or 20%, for IPT and TB treatment.

#### Decision rules

The lack of cost-effectiveness data comparing these screening strategies, especially in the context of IPT, implies knowing the least costly strategy is essential. The WHO recommends in resource constrained countries interventions where the value of a unit of health gain is less than the gross national income (GNI) per capita as being ‘very cost-effective’, values less than three times being ‘cost-effective’ [64]. The GNI per capita inflated to 2010 for the sub-Saharan region is 2167 US Dollars [34], [65], and therefore our maximum willingness to pay (WTP) thresholds were 2167 US Dollars (λ_1_) and 6500 US Dollars (λ_2_).

## Results

The findings of the base-case analysis are shown in Table 2. The mean cost to screen and provide either TB treatment or IPT ranged from $169 to $325 per individual. The least costly strategy involved screening with sputum microscopy (Strategy 5), and the most effective strategy involved screening for the presence of any classical TB symptoms (Strategy 2). The incremental cost utility analysis shows that Strategy 1 is strongly dominated, whilst strategies 3, 4, 6 and 7 are weakly dominated and therefore none of these strategies would be recommended in terms of cost-effectiveness. If our willingness to pay for an extra QALY were zero then Strategy 5 would be the most cost-effective. In comparison to Strategy 5 the other strategies would not be considered cost-effective at either of our threshold ICERs. Strategy 8 would be cost-effective at a threshold ICER of $7,800/QALY. Strategy 9 becomes optimal at a threshold ICER of $13,600/QALY and Strategy 2 at thresholds over $24,500/QALY. Comparison of the effectiveness of the non-dominated strategies, 5, 8, 9 and 2, reveals 75.8%, 90.2%, 92.8% and 94.0% of individuals with active TB will successfully complete TB treatment, respectively, whilst 1.9%, 16.3%, 23.7% and 53.1% of individuals without active TB will receive TB treatment, respectively. In comparison to Strategy 5, the incremental cost for an additional individual with active TB to successfully be treated was $4,700, $12,100 and $74, 700 for strategies 8, 9 and 2, respectively, whilst the incremental cost to prevent an additional individual dying from active TB, or as a consequence of adverse drug reactions to TB treatment or IPT, was $5,700, $11,000 and $21,900, respectively.

**Table 2:** Findings from the base-case model.

	Mean Cost ($)	Mean QALY's	% of individuals wrongly treated for TB	% of individuals with active TB completing treatment	Incremental Analysis				
					Cost ($)	QALYs	$/QALY	$/Individual Successfully treated for TB*	S/Death averted**
**Strategy 5**Sputum on all	169	1.135	1.9	75.8	-	-	-	-	-
**Strategy 3**Two or more Classic symptoms	191	1.136	11.9	76.3	22.0	0.001	ED	ED	ED
**Strategy 7**Symptom +/− Sputum	207	1.137	14.3	77.9	16.1	0.001	ED	ED	ED
**Strategy 4**CXR on all	221	1.142	15.4	88.7	13.5	0.005	ED	ED	ED
**Strategy 1**Chronic cough>2 weeks	221	1.137	20.1	77.6	0.6	−0.005	D	D	D
**Strategy 8**Symptom +/− Sputum +/− CXR	225	1.143	16.3	90.2	4.3	0.001	7,775	4,656	5,675
**Strategy 6**Symptom +/− CXR	241	1.143	22.8	90.1	16.9	<0.001	ED	ED	ED
**Strategy 9**Symptom +/− CXR +/− Sputum	251	1.144	23.7	92.8	9.3	0.001	13,552	12,145	11,001
**Strategy 2:**Any Classic symptom	325	1.147	53.1	94.0	73.9	0.003	24,376	74,710	21,870

QALY: quality-adjusted life year.

D: dominated strategy (one or more alternative strategies are cheaper and more effective).

ED: extended dominance (one or more alternative strategies more expensive but have lower ICER).

$ 2010 US Dollars.

*Incremental cost per additional individual with active TB who successfully completes TB treatment course.

**Incremental cost per death averted (either from active TB, adverse drug reaction to TB treatment or IPT).

### Sensitivity analysis and Value of information

**Figure 3:**
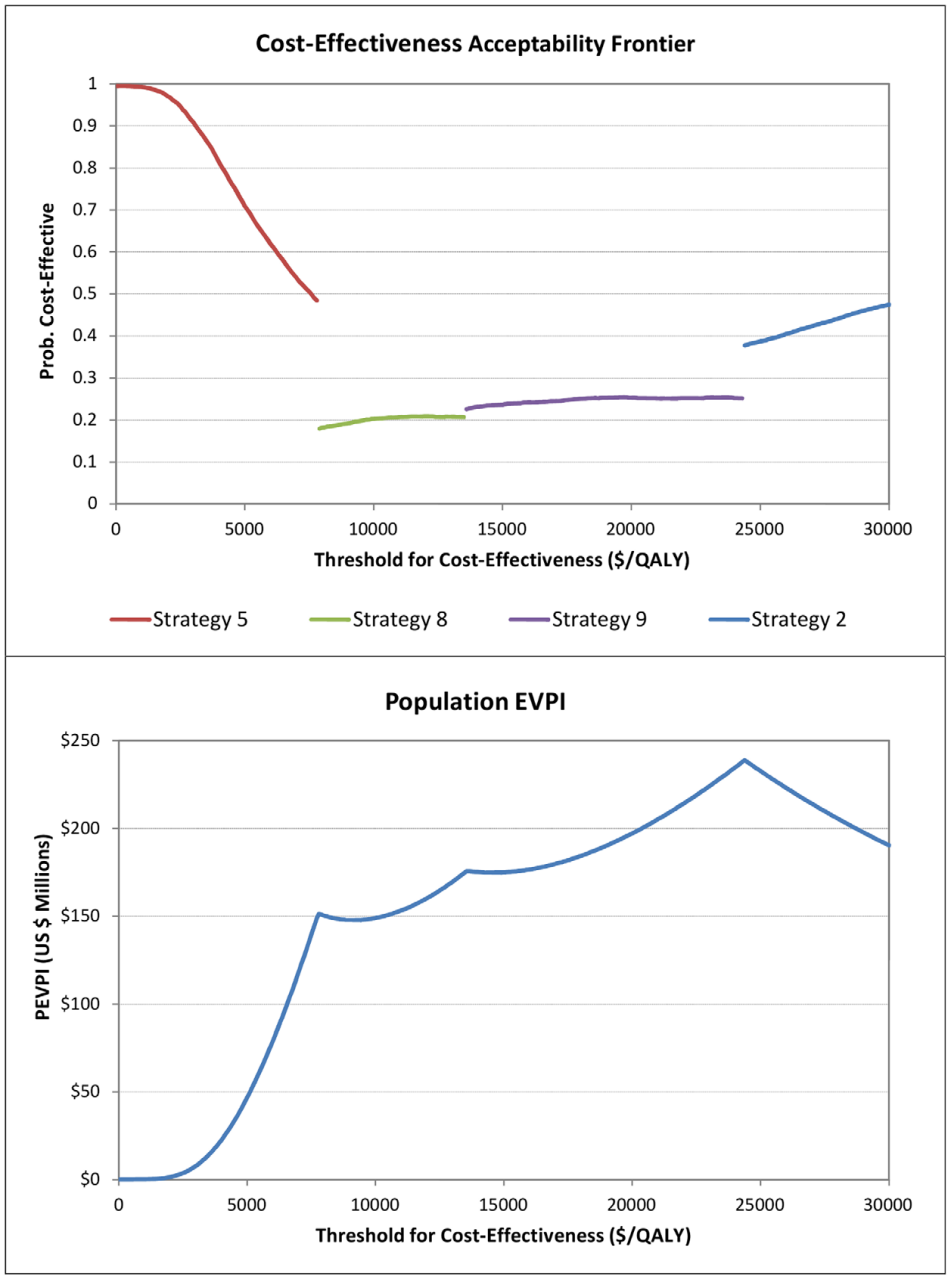
CEAF and EVPI curves for the base case model.

The optimal strategies are shown on a cost-effectiveness frontier (CEAF). Cost-effectiveness acceptability frontiers provide a method of representing the optimal decision at differing threshold ICERs in the context of multiple alternatives [66]. Figure 3 shows the CEAF and highlights that whilst Strategy 8 is the optimal decision at the threshold ICER of $7,800/QALY, we are only 18% certain of this. At our very cost-effective (λ_1_) and cost-effective thresholds (λ_2_), the probability that Strategy 5 is optimal is 0.96 and 0.58 respectively, the probability that Strategy 8 is optimal is 0.01 and 0.15 respectively, the probability that Strategy 9 is optimal is <0.01 and 0.09 respectively, whilst the probability Strategy 2 is optimal is <0.01 and 0.01 respectively.

We calculated the EVPI and EVPPI for current and future populations [54], assuming an annual HIV incidence in sub-Saharan Africa of 1.5 million [1], and a conservative estimate of 5 years for the lifetime of the decision problem. Figure 3 displays the population EVPI (PEVPI) at increasing threshold ICERs, and Table 3 shows the PEVPI and population EVPPI (PEVPPI) at our two WTP thresholds. The PEVPI peaks at a threshold of about $24,400/QALY, the point where the optimal strategy changes from Strategy 9 to 2. The PEVPI at our two cost-effectiveness threshold ICERs of λ_1_ and λ_2_ was about $2 million and $98 million respectively. The PEVPPI output suggests improving our estimate of the prevalence of TB and LTBI in our target population is likely to provide the greatest payoff. Further research into the costs of the different screening strategies would be of more value than further research into their diagnostic accuracy, whilst further research in TB treatment is expected to provide more value than research in IPT treatment.

**Table 3:** Findings from population EVPI and EVPPI analysis.

	PEVPI/PEVPPI at WTP = λ_1_(Million $)	PEVPI/PEVPPI at WTP = λ_2_(Million $)
**Base-case analysis**	**2.0**	**97.7**
Cost of Screening Strategy	1.2	12.2
Sensitivity and Specificity of Screening Strategy	<0.1	8.9
Prevalence of active TB and latent TB infection	1.7	51.5
TB treatment costs and outcomes	0.7	9.0
IPT treatment costs and outcomes	0.4	0.4
Health state utilities	0.0	0.0

$ 2010 US Dollars.

**Figure 4:**
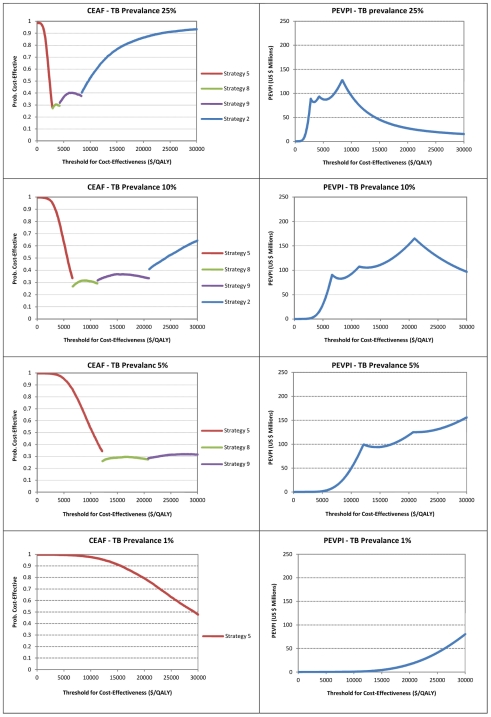
CEAF and PEVPI curves at different TB prevalence.

We explored the effect of TB prevalence on the optimal decision and value of further research. We re-ran the model keeping the TB prevalence constant at 1%, 5%, 10% or 25%; Figure 4 shows the CEAF and EVPI curves. As the true TB prevalence decreases from 25% to 1%, the threshold ICER at which the other strategies becomes optimal increases, whilst the value of further research at our two threshold ICERs decreases. If the true TB prevalence were less than 5%, none of the other strategies would be optimal at threshold ICERs below $10,000/QALY.

### Sensitivity analysis: Alternative model structures

**Figure 5:**
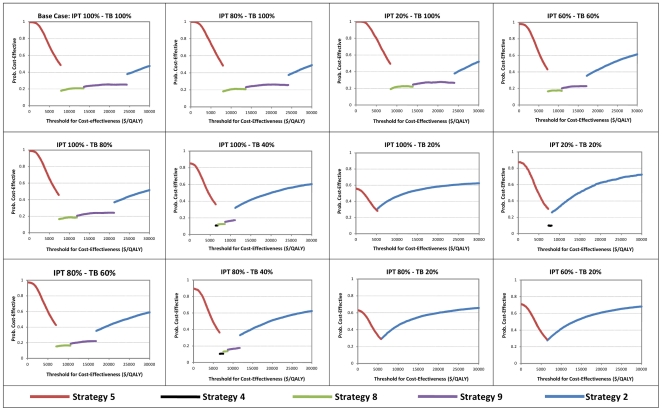
CEAF curves for different TB treatment and IPT completion probabilities.

We explored the effect of LTFU on the optimal decision. Figure 5 shows the CEAF for a range of completion probabilities for TB treatment and IPT. Strategy 5 remains optimal at threshold ICERs up to $5,200/QALY irrespective of the completion probabilities of IPT or TB treatment. The only scenarios where Strategy 5 is not optimal at our cost-effective threshold (λ_2_) is where TB treatment completion falls below 40% and IPT completion is 100%, or where TB completion falls below 20% and IPT completion is higher than 80%. In these scenarios, either strategy 2 or 4 were optimal. Where completion probabilities of IPT and TB treatment are comparable, strategy 5 remains optimal at our cost-effective threshold (λ_2_).

## Discussion

We modelled the costs and consequences of different TB screening strategies that could be employed if all individuals testing HIV positive in sub-Saharan Africa were to be screened for active TB prior to starting IPT. The findings suggest the least costly strategy involves screening with sputum microscopy (Strategy 5). At the WHO threshold ICERs, the other strategies evaluated would not be considered cost-effective in comparison. The mean cost per individual screened and treated for either on TB or LTBI with Strategy 5 was $169, and comparable to previous findings [29]–[31]. The VOI analysis suggests further research would be of value, although possibly only at our higher threshold ICER (λ_2_). The value of knowing the true TB prevalence in the population to be screened is especially important. Not only does it impact on our optimal decision, it also affects whether subsequent research into the other parameters would be cost-effective (Figure 3).

The model considers screening for active TB and providing IPT as a single decision problem. Whilst screening strategies with higher sensitivities are more effective, as greater proportion of individuals with active TB successfully complete treatment, it is their relative specificity that determined cost-effectiveness. The majority of the costs incurred are as a result of providing either TB treatment or IPT. As providing TB treatment is about four times as costly as IPT, falsely starting TB treatment incurs considerable cost with no benefit. Screening for the presence of any of the classical symptoms of TB, Strategy 2, has been shown to be a reliable method of excluding TB prior to starting IPT [67], especially as there are concerns that starting INH in individuals falsely excluded for TB may increase the burden of multi-drug resistant TB [68]. The model suggests that whilst this was the optimal symptom based screening strategy and the most effective, it would not be cost-effective at our threshold ICERs.

Investigating TB suspects with sputum microscopy and/or CXR was cost-effective, albeit at higher threshold ICERs, only if both investigations were performed. As with previous findings, the more cost-effective option would be to screen symptomatic individuals with sputum followed by CXR (Strategy 8) rather than CXR followed by sputum (Strategy 9) [28], [69]. The incremental analysis suggests Strategy 8 is cost-effective at a threshold ICER of US$ 7,800/QALY. Although, at this threshold ICER we are only 18% confident this would be the optimal decision, whilst we are more or less 100% confident choosing Strategy 5 is the least costly strategy. Whilst there is uncertainty about the role of CXR in the screening for TB in HIV infected individuals [70]–[72], the model suggests CXR does have value but only in conjunction with sputum microscopy in TB suspects.

The underlying TB prevalence has a deterministic effect on the optimal decision. If the true TB prevalence in the population screened is lower than 5%, we are almost certain that the optimal decision would be to screen everyone with sputum microscopy, with alternate strategies cost-effective at very high threshold ICERs. At prevalences higher than 10%, other strategies become cost-effective at affordable levels. In practice the prevalence of TB varies on the setting where ICF is employed [40] and therefore it may be rational to employ different screening strategies depending on the setting.

We explored the consequences of LTFU and found Strategy 5 remains optimal at threshold ICER's up to $5,200/QALY irrespective of the degree of LTFU. If, as the literature suggests [43], [58]–[63], LTFU in those started on IPT and TB treatment is comparable, Strategy 5 remains optimal at our higher threshold ICER (λ_2_). The only scenario in which LTFU alters the optimal strategy at our higher threshold ICER (λ_2_) is where LTFU amongst those started on TB treatment is high (≥60%) and LTFU amongst those started in IPT is low (≤20%). We made the assumption that LTFU was dependent on the treatment given. However, there may be greater attrition for strategies that require more clinic visits. This is likely to have a greater impact on Strategies 6–8 as they require more clinic visits.

Recently it has been highlighted that the lack of operational research in tuberculosis may be a consequence of determining what the research priorities are [73]. VOI analysis achieves this by ensuring future research is directed to answering decision problems faced by policy makers. It offers significant advantages, for example, it draws attention to the finding that further research into the costs of the screening strategies offers more value than research into their diagnostic accuracy. It informs us that whist there are concerns regarding the validity of the available utility data [48], further research into determining utility scores for HIV positive individuals with TB or LTBI will have little impact on this decision. This is one of the first studies to use VOI analysis in resource-constrained settings, and should encourage its continued use.

The analysis was carried out in accordance with WHO guidelines [35]. We undertook a formal systematic review and meta-analysis to evaluate the nine different screening strategies, and used cost data standardised to sub-Saharan Africa to aide generalisability. The main limitations are that it uses a static model and fails to consider consequences of developing INH resistance. Whilst the model does not consider the consequences of ICF and IPT on TB transmission, evidence suggests it is the HIV negative TB cases that drive TB prevalence [74], and therefore unlikely the TB screening strategies will have differing levels of impact on TB prevalence. Any impact is unlikely to alter the fact that screening everyone with sputum microscopy will remain optimal, as it is smear positive TB that drives TB transmission. We did not consider the development of INH resistance in those falsely excluded for active TB. Currently, the evidence suggests no increased risk [75], [76]. If future research reveals increased risk, strategies associated with higher sensitivities are likely to be cost-effective at lower thresholds than found in the model.

The low sensitivity of sputum microscopy in HIV infected individuals is felt to limit its use [72], but its high specificity makes it a cost-effective option for programs wishing to scale up both ICF and IPT in their HIV infected population, especially if the true TB prevalence is below 5%. In settings where the underlying TB prevalence is known to be higher, it is likely that a combination of symptom screening and diagnostic procedures would be cost-effective.

## Supporting Information

Figure S1 **Markov model: True Positive.**
(JPG)

Figure S2 **Markov model: False Positive.**
(JPG)

Figure S3 **Markov model: True Negative.**
(JPG)

Figure S4 **Markov model: False Negative.**
(JPG)
