# Supplementary figures and images for: Intensive Case Finding and Isoniazid Preventative Therapy in HIV Infected Individuals in Africa: Economic Model and Value of Information Analysis
Source: PLoS One. 2012 Jan 23;7(1):e30457. doi: 10.1371/journal.pone.0030457 (PMC3264596; doi:10.1371/journal.pone.0030457)

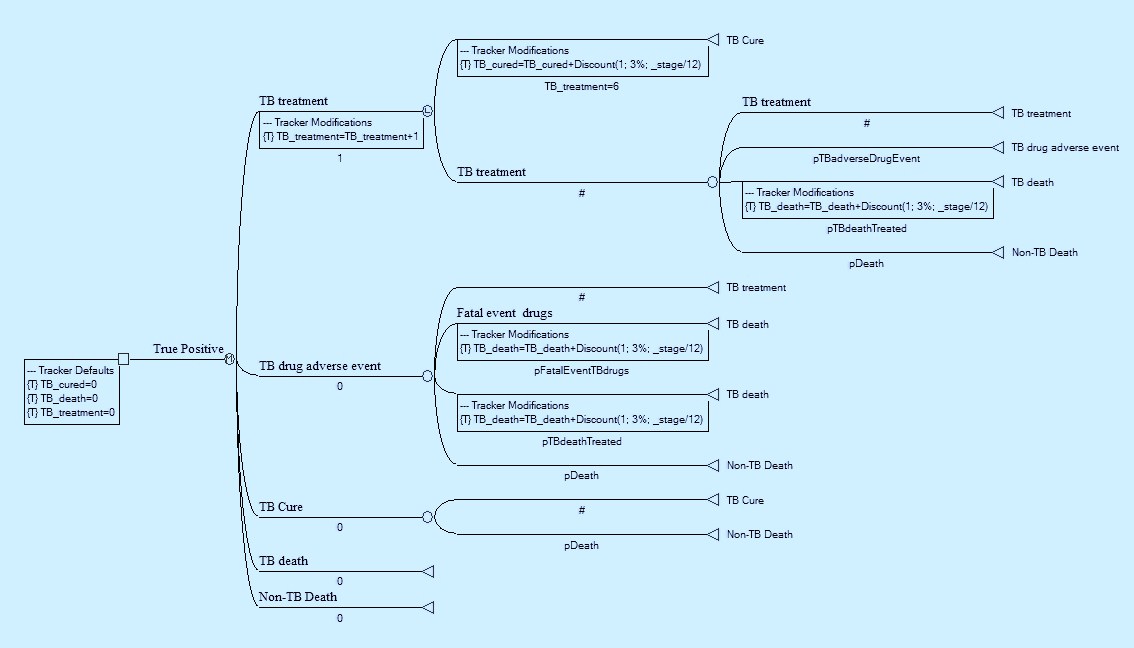

Supplement: Figure S1 — Markov model: True Positive. (JPG) [file pone.0030457.s001.jpg]

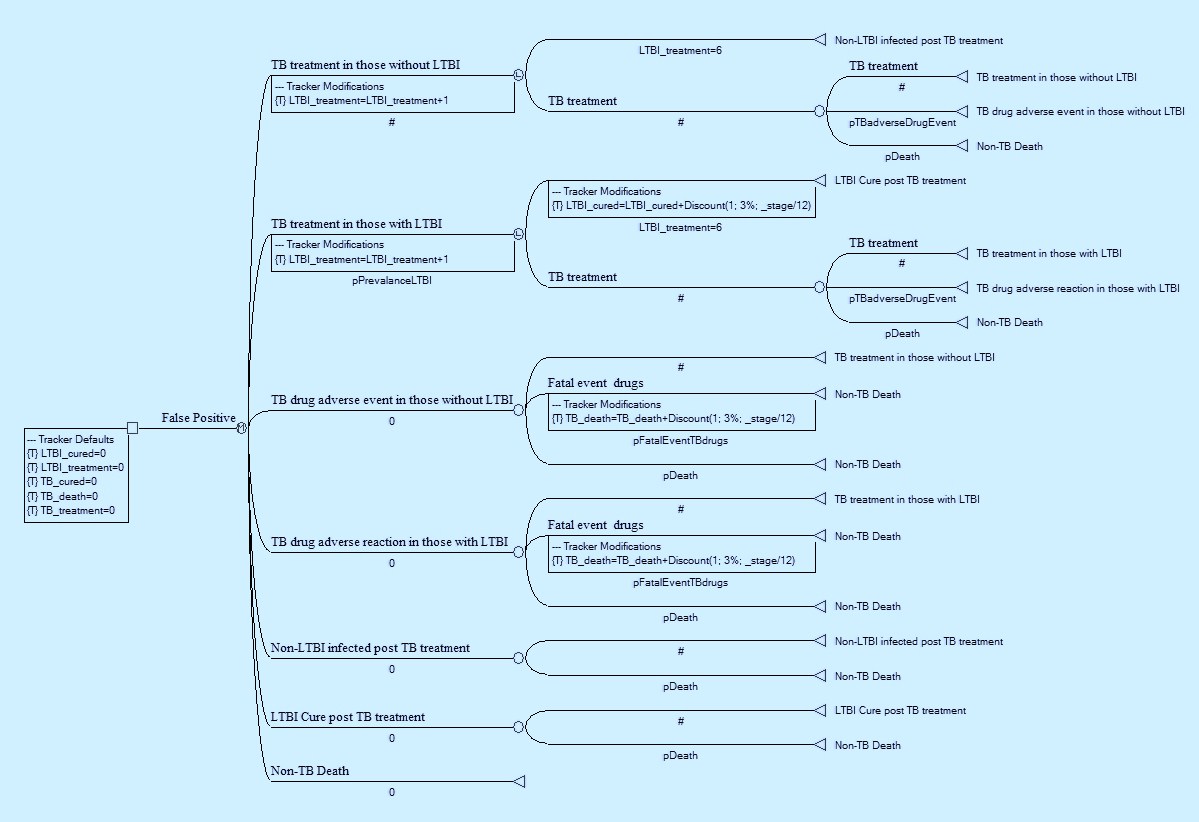

Supplement: Figure S2 — Markov model: False Positive. (JPG) [file pone.0030457.s002.jpg]

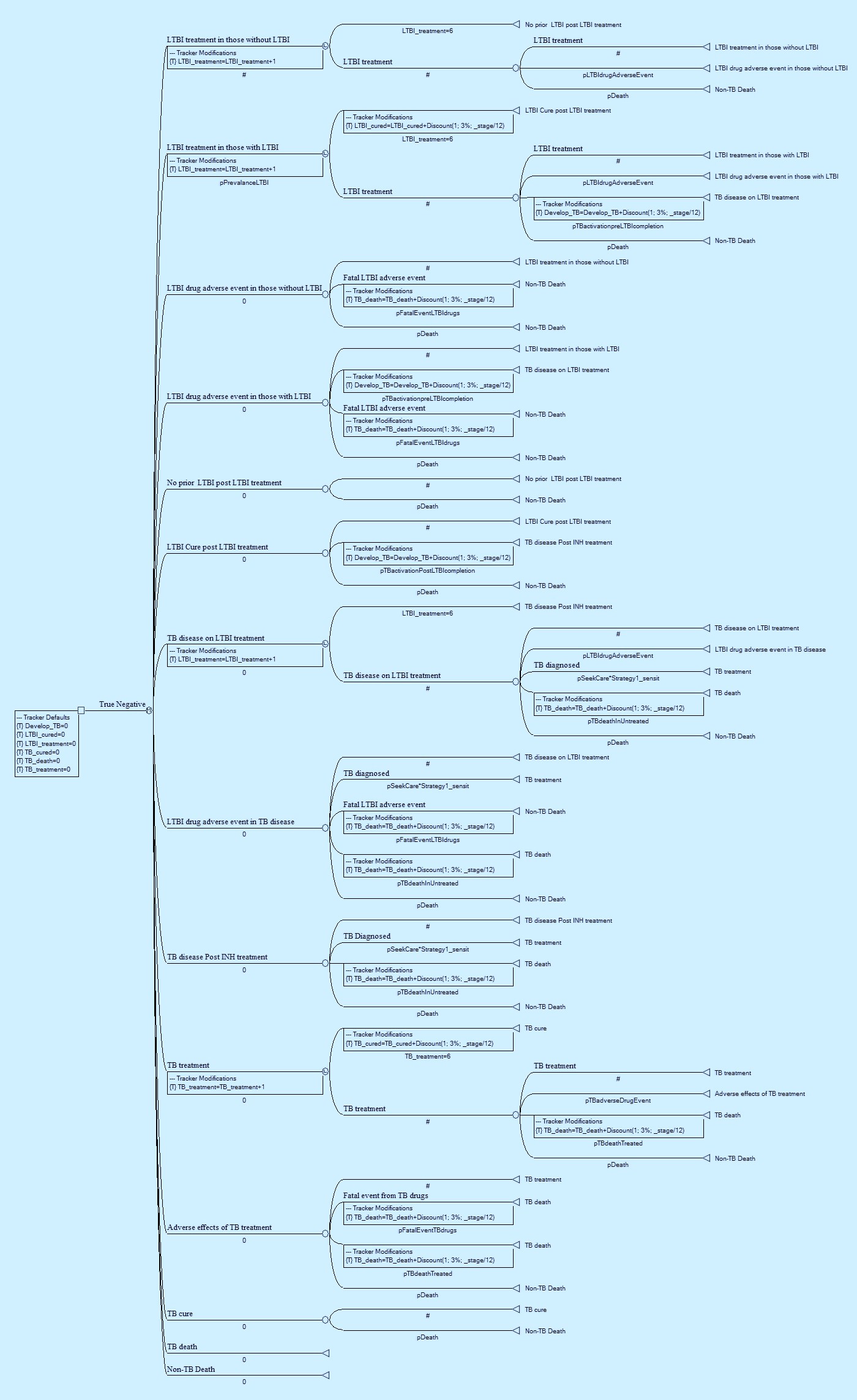

Supplement: Figure S3 — Markov model: True Negative. (JPG) [file pone.0030457.s003.jpg]

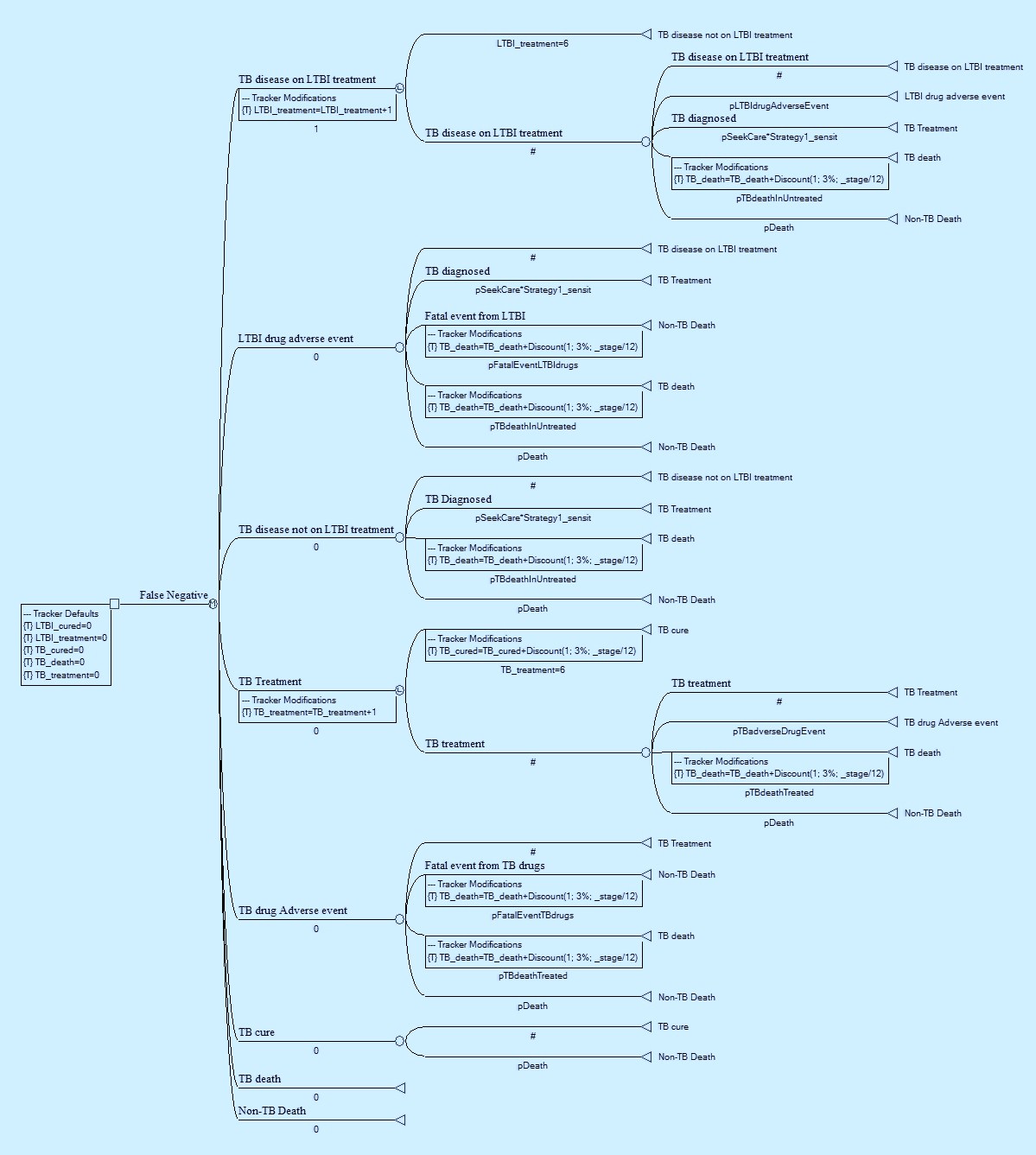

Supplement: Figure S4 — Markov model: False Negative. (JPG) [file pone.0030457.s004.jpg]
